# Application of *Stichopus hermanni* Nanoparticle Gel in the Healing of Traumatic Ulcers

**DOI:** 10.1055/s-0042-1759884

**Published:** 2023-01-23

**Authors:** Rima Parwati Sari, Debora Intan Dewi Larashati, Clarissa Aldiana, Nafi'ah Nafi'ah, Dian Widya Damaiyanti, Atik Kurniawati

**Affiliations:** 1 Department of Oral Biology, Faculty of Dentistry, Universitas Hang Tuah, Surabaya, Indonesia; 2 Department of Oral Medicine, Faculty of Dentistry, Universitas Hang Tuah, Surabaya, Indonesia; 3 Department of Oral Biology, Faculty of Dentistry, University of Jember, Jember, Indonesia

**Keywords:** *Stichopus herrmanni*, nanoparticle, traumatic ulcers, COL-1 expression

## Abstract

**Objective** The aim of this research was to investigate the use of *Stichopus herrmanni* nanoparticle gel on the ulcer healing process by observing blood vessels, fibroblasts, and Collagen type-I (COL-1) expression on the 4 and 7th days after trauma.

**Materials and Methods** Gold sea cucumber ( *Stichopus herrmanni* ) powder was processed by freeze-drying method, then by high-energy milling to form nanoparticle size, and then with CMC 2% to make hydrogel. Traumatic ulcers were formed by induction using a burner. Five groups of male Wistar rats, each consisting of six tails, were divided into a negative control group that was given a placebo, the positive control group was given 0.2% hyaluronic acid, and the treatment group was given gold sea cucumbers with concentrations of 0.135, 0.27, and 0.54% (SH1-SH2-SH3). Fibroblast and blood vessels were examined with hematoxylin-eosin on day 3 and 7, while COL-1 expression was examined with immunohistochemistry on day 7. The rats' mucosa was taken on the 3rd and 7th days after the traumatic ulcer was formed.

**Statistical Analysis** The data were analyzed using a one-way analysis of variance followed by a post-hoc test with a *p* less than 0.05.

**Results** Nanoparticles gel freeze-drying of *Stichopus herrmanni* increased blood vessels on day 3. Angiogenesis continued to occur, which resulted in increased fibroblast and COL-1 expression on day 7.

**Conclusions** The application of *Stichopus herrmanni* nanoparticle gel at 0.27% effectively increased the number of blood vessels, fibroblasts, and COL-1 expression in healing traumatic ulcers.

## Introduction

Traumatic ulcers are the most common oral lesion. The most common causes of ulcers are mechanical, chemical, heat trauma, thermal or temperature, and radiation. 1 Clinical feature of ulcers is generally oval with a yellowish-white necrotic center with prominent margins. It is a reddish, painful area confined to the damaged area, measuring approximately 5 to 10 mm, and progresses to spontaneous healing in a period that can vary from 10 to 14 days without leaving a scar. 2

The wound healing process consists of a hemostatic phase, an inflammatory phase, a proliferative/reparative phase, and a maturation/remodeling phase. 3 In the hemostatic phase, there is a formation of a clot that supports immune cell infiltration and migration and initiates tissue repair pathways. The inflammatory phase is the initial stage of the molecular activation process involving immune cells, the secretion of alarmin signals, and the activation of keratinocytes and fibroblasts. In the proliferative phase, keratinocytes, fibroblasts, and endothelium migrate and proliferate, initiating the formation of de novo granulation tissue. In remodeling (maturation) phase, there is a change in the collagen matrix from collagen III to collagen I, reorganization and rearrangement of the extracellular matrix, endothelial cell migration, and endothelial cell proliferation. The formation of new blood vessels or angiogenesis is crucial in wound healing because blood vessels channel nutrients, various healing mediators, and membrane metabolites. The formation of blood vessels is seen in the proliferation process as early as 4 to 5 days after wound formation. 4 5 Angiogenesis in wound healing involves various stages, namely the initiation process, the release of protease enzymes from activated endothelial cells, and degradation of the basement membrane in the form of an extracellular matrix, followed by the formation of capillaries, parallel capillary anastomosis, and ended with the formation of new membranes to meet the needs of the tissue and also to accelerate the healing process. 4 6

A topical and systemic drug is needed in traumatic ulcer therapy, which is not only to reduce pain (symptomatic) but also to speed up the healing process. One of the curative drugs used topically, 0.2% hyaluronic acid (HA), has been started to be used as a drug for treating traumatic ulcers. One of the curative drugs, 0.2% HA, has been started to be used as a drug for treating traumatic ulcers. HA plays a role in tissue healing by activating the inflammatory response, triggering cell proliferation, migration and angiogenesis. It triggers re-epithelialization through the proliferation of basal keratinocytes and reduces the placement of collagen and scar tissue. 7 The gold sea cucumber ( *Stichopus herrmanii* ) is one of the marines that contains much HA (75.7%). In this study, the whole meat of *Stichopus herrmanni* was freeze-dried. 8

Nanoparticle technology has become a new trend in developing drug delivery systems. Particles or globules at the nanometer scale have distinctive physical properties compared with particles at larger sizes, especially in improving the delivery quality of drug compounds. 9 Another advantage of nanoparticle technology is that it can increase drug stability, can reach specific targets in cells or tissues, and can modify the release, thus opening the opportunity to produce a perfect delivery system. 10

This study aimed to determine the effect of topical administration of nanoparticle-sized gold sea cucumber ( *Stichopus herrmanni* ) gel on increasing the number of blood vessels, fibroblasts, and COL-1 expression on the healing process of oral ulcers.

## Materials and Methods

### Preparation of *Stichopus herrmanni* Nanoparticle with Freeze-Drying Method

The golden sea cucumber meat, separated from the internal organs, was washed with sterile distilled water. The golden sea cucumbers were crushed using a blender of 1:2 (w/v). The mixture was put in the freezer at −80°C for 24 hours. The following drying method was the freeze-drying method with a pressure of 20 Pa in a temperature of 4°C until a dry preparation was formed and blended to become a powder. The following process was the making of the gold sea cucumbers into nanoparticle size, using high-energy milling Elliptical 3D Motion by Nanotech Indonesia. 11 The gold sea cucumber powder was sifted with a mesh of 50 is and was divided into five groups. Group 1 was milled with ball milling with a diameter of 5 mm for 20 minutes with a working time of 10 minutes of milling and 10 minutes of rest with two repetitions. Group 2 was treated in the same way as group 1, but repeated three times, while Group 3 was also treated in the same way with four repetitions. Group 4 received the same treatment but for 30 minutes with three repetitions. Group 5 was treated as the same as group 4, but in the third repetition the ball milling was replaced with that in a size of 2 mm. The gold sea cucumber powder was put into a cylindrical capsule with ball milling with a ratio of 1:10 (w/w) gold sea cucumber powder. The tool must be rested when the following process was performed according to the length of work, and the powder was stirred to prevent clumping.

### Particle Size Analyzer Test

One gram of gold sea cucumber powder was dissolved in 8 mL of 1% acetic acid and sonicated for 10 minutes to obtain a homogeneous sample. The sample was placed in a suitable single-use plastic cuvette, then measured with a beta nanoparticle analyzer (VASCO™, Cordouan Technologies, France) times per group. 12 The optimal attenuator slit width was 6 to 8. If the attenuator gap showed the number 6, the sample needed to be dissolved, while if the attenuator showed number 8, the sample substance needed to be added again. The prostate-specific antigen (PSA) test results were tabulated and analyzed using the one-way analysis of variance (ANOVA) and Tukey Honest Significant Difference (HSD) post-hoc tests.

### Preparation of *Stichopus herrmanni* Nanoparticle Gel

This material was made by mixing 0.27 g of dried gold sea cucumber into 100 mL of 1% acetic acid and stirred for 3 hours using a stirrer at room temperature. The next step is was the addition of 10% NaOH until the pH became 7. The addition of 2 g of NaCMC is was performed to form an Orabase gel preparation so that the viscosity was medium and more stable. In addition, the preparation of the Orabase gel dosage form was performed so that it could adhere well to the oral mucosa, giving time for the drug to be absorbed more and increasing the drug's duration of action. 13

### Preclinical Test to the Experimental Animals

The experimental animals used in this research were *Rattus norvergicus* aged approximately 4 months with body weights of 200 to 300 g. This research was conducted after obtaining permission from the ethical commission of the FKG UHT, Surabaya, with No. EC/031/KEPK-FKGUHT/XII/2021. The study was started by acclimatizing the experimental animals for 7 days. The day before the experiment, the *Rattus norvergicus* were fasted overnight. The experiment was started by weighing the rats. Anesthesia was administered using Ketamine 10% injection (KETAMINE 10% Inj, Kepro Pharmacy, Holland) at a dose of 0.1 cc/100 g body weight and Xylazine 2% injection (Xyla, Interchemie, Netherland) at 0.01 cc/100 g body weight intramuscularly in the upper thigh. 14 Furthermore, the lower labial mucosa in the animals was smeared with 1% povidone-iodine. A cement stopper hand instrument with a 2 mm diameter was heated over a flame burnisher for 1 minute, then tapped on the labial mucosa of Wistar rats for 0.25 seconds. Traumatic ulcers developed after 24 hours as yellowish-white ulcers surrounded by an erythematous halo. After confirming ulcers formed, the rats were randomly divided into five groups, namely the negative control group given a placebo, the HA group given 0.2% Hyaluronic acid gel (Gengigel, Ricerfarma s.r.l., Italia), and SH1–3 given *Stichopus herrmanii* nanoparticle gel with a concentration of 0.135%, 0 0.27%, and 0.54%

### Histological Study

Four and seven days after the application of the gel on the ulcers, the animals were sacrificed, and the labial mucosal preparations were taken and put into 10% formaldehyde buffer solution for tissue fixation. Labial mucosal specimens were made in a sagittal section and stained with hematoxylin-eosin and immunohistochemistry with monoclonal anti-COL-1 (ab88147, abcam, United States). After that, the number of blood vessels and fibroblasts were observed on the 4th and 7th day, and the expression of COL-1 was observed on the 7th day by light microscopy (Olympus CX21, Olympus IMS, Japan) at 100X and 400X magnification. Furthermore, data tabulation and statistical analysis were performed with one-way ANOVA followed by the Tukey HSD test.

## Results

### PSA Test

**Fig. 1:**
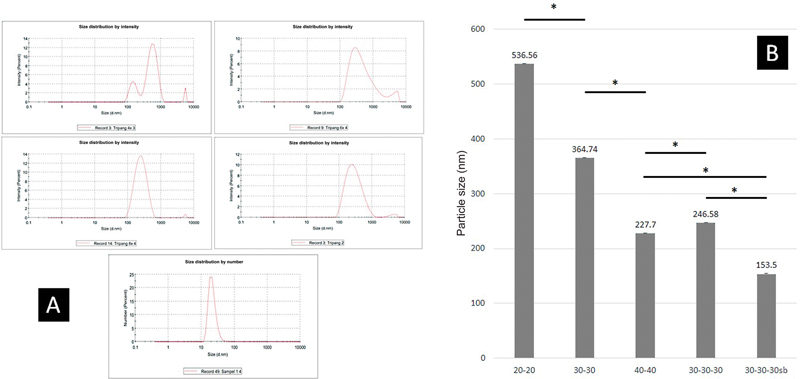
Particle size distribution and average prostate-specific antigen (PSA) test results of *Stichopus herrmanni* powder in several groups divided according to the length and diameter size of stainless ball milling. ( **A** ) These images show the particle distribution successfully measured by the PSA tool, where the use of ball milling with a small diameter (2 mm) produces the expected nanoparticle size. ( **B** ) The figure shows that the longer the time used for grinding, the smaller the golden sea cucumber powder particles will be. Ball milling size also affects the resulting particle size. The graph shows a significant difference (*) in milling using a stainless ball (5 mm) every 20 minutes, 30 minutes, and 40 minutes with two milling times. Likewise, the difference in the particles produced in the milling process is carried out with smaller ball size stainless which is more significant in producing smaller particles (2 mm) than the larger stainless ball sizes (5 mm).

The PSA test data showed that the two to three times pounding process with different ball milling variations affected the particle size of the gold sea cucumber powder. These data were confirmed by the one-way ANOVA test analysis, which showed a significant difference where the pounding process two times, namely 30 minutes of working time and 30 minutes of rest time, produced particle in a size of 153.5 nm. The profile of the particle size distribution and the average of the PSA test results can be seen in Fig. 1 .

### Fibroblast Count in Ulcer Healing on the Fourth and Seventh Days

**Fig. 2:**
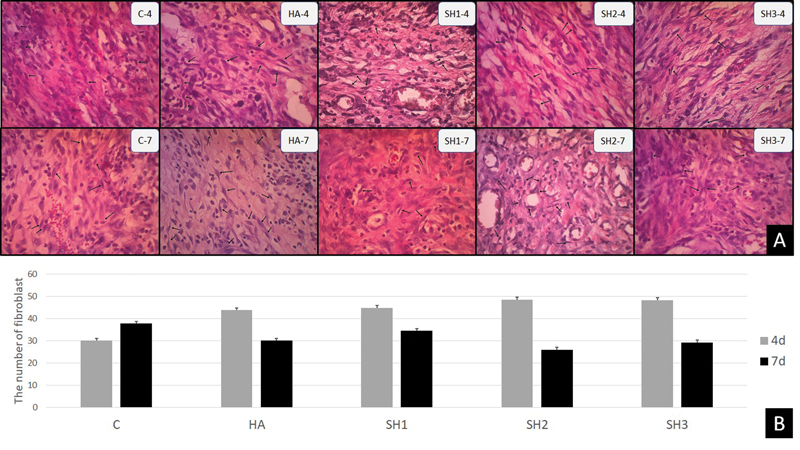
Histological section of fibroblast with the application of Stichopus herrmanni nanoparticles gel. ( **A** ) Observation using a light microscope at 400x magnification. Pathological conditions are shown in the control group (K-), hyaluronic acid (HA) (K þ), and P1–3 (SH1–3) in 4 days. ( **B** ) This figure shows that the highest number of fibroblasts was in the SH2 group on day 4, but on day 7, the highest number of fibroblasts was in the control group.

The number of fibroblasts was observed on the histopathological labial mucosa with HE staining and 400X magnification. The results of the observations through a light microscope with 400X magnification can be seen in Fig. 2A . Description analysis showed that the group given with *Stichopus herrmanni* nanoparticle gel in a concentration of 0.27% (SH2) had the highest number of fibroblasts on day 4, while the lowest was in the control group ( Table 1 , Fig. 2B ). The number of fibroblasts on the 7th day decreased compared with that on the 4th day but this did not occur in the control group.

**Table 1:** Data analysis fibroblast, blood vessels, and COL-1 expression on myofibroblast in labial mucous on the 4th and 7th days after trauma

Groups	Fibroblast									Blood vessel									COL-1			
	4 d				7 d				*p* -Value	4 d				7 d				*p* -Value	7d			
C	30	±	3.92	^ a^	37.75	±	4.35	^ a^	0.664	14.75	±	1.71	^ a^	24.5	±	2.65	^ a^	0.021*	13.75	±	4.19	^a^
HA	43.75	±	8.69	^ ab^	30	±	3.74	^ a^	0.53	29.25	±	2.87	^ b^	39	±	3.65	^ b^	0.021*	31.25	±	12.92	^ab^
SH1	44.75	±	4.57	^ b^	34.5	±	5.45	^ a^	0.297	33.5	±	3.11	^ b^	42.5	±	2.87	^ bc^	0.043*	21.25	±	10.34	^ab^
SH2	48.5	±	5.45	^ b^	26	±	4.32	^ a^	0.02*	38	±	3.37	^ b^	49	±	2.94	^ c^	0.006*	36.5	±	6.61	^b^
SH3	48.25	±	10.24	^ b^	29.25	±	1.5	^ a^	0.02*	36.5	±	6.76	^ b^	48.25	±	4.35	^ c^	0.003*	33.5	±	3.87	^b^

NOTE : a,b,c,d Difference between the groups with significance level of 5% ( *p* < 0,05).

Abbreviations: HA, Hyaluronic Acid ; COL-1, Collagen type-I

The multiple comparison Tukey HSD test results in Table 1 , which was based on the type of treatment, on day 4 showed a significant difference between the control group and all SH groups, while the control and HA groups did not show any significant differences. Based on the length of treatment on the 4th and 7th days, no significant difference occurred in the control group, HA and SH1, while a significant difference occurred in the SH2 and SH3 groups. On the 7th day, the *p* -value of the Tukey HSD test showed no significant difference in all groups.

### Blood Vessels Count in Ulcer Healing on the Fourth and Seventh Days

**Fig. 3:**
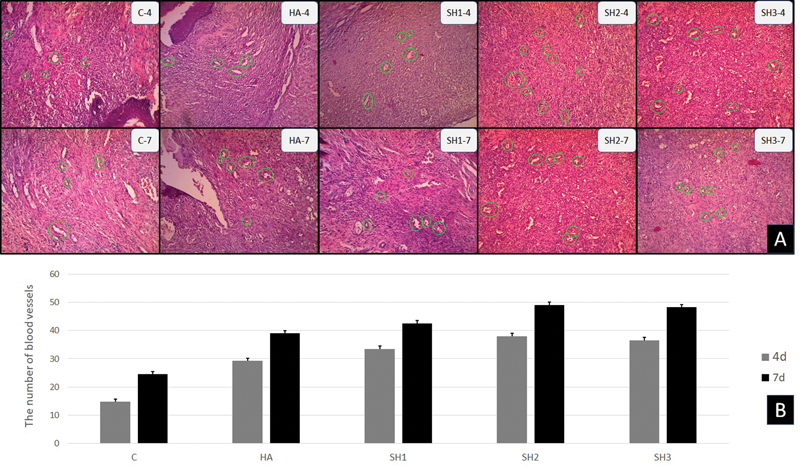
Histological section of blood vessels with the application of *Stichopus herrmanni* nanoparticles gel **.** Observation using a light microscope at 100x magnification. Pathological conditions in the control groups (K-), hyaluronic acid (HA) (K + ), and P1–3 (SH1–3) in 4 days ( **A** ). This figure shows that the highest number of blood vessels on the 4th day was in the SH2–3 group on the 4th day, and the same was repeated on the 7th day ( **B** ) **.**

Observations on the same preparation were also performed to count the number of blood vessels, but observations were made with 100X magnification ( Fig. 3A ). The results of the descriptive analysis showed that on the 4th day of observation, the highest number of blood vessels in the SH1 group continued to increase on the 7th day. An increase also occurred in all groups. The results of the observation on the number of blood vessels can be seen in Table 1 and Fig. 3B .

The results of the multiple comparison Tukey HSD test are presented in Table 1 . Based on the type of treatment, the results on day 4 and day 7 showed a significant difference between the control group and all other groups. The results on day 7 were also significantly different between control and other treatment groups. The HA group and the SH2–3 group also showed significant differences. The nonsignificant difference was only shown by the HA and SH1 groups.

### Observation of COL-1 Expression in Ulcer Healing on Day 7

**Fig. 4:**
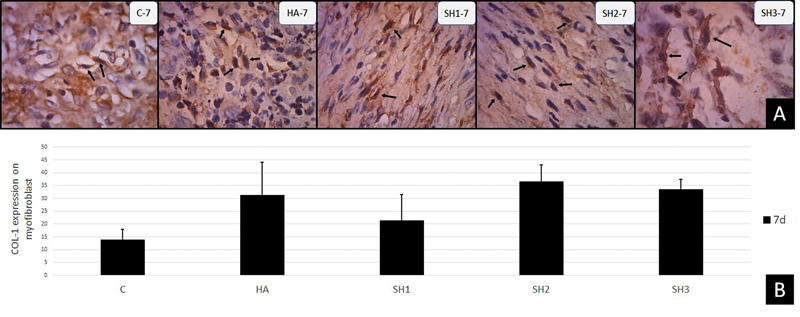
Histological section of COL-1 expression with application of *Stichopus herrmanni* nanoparticles gel. ( **A** ) Pathological conditions in control group (K-), hyaluronic acid (HA) (K + ), and P1-3 (SH1-3) on day 4. Observation using a light microscope at 400x magnification. ( **B** ) The increase in COL-1 expression was seen most in the SH2 group, followed by the SH3 and HA groups.

Results of observations of COL-1 expression in myofibroblast cells can be seen in Table 1 and Fig. 4A . In each group, data on positive expression of COL-1 were obtained from the observation of labial mucosal myofibroblast cells by immunohistochemical method. The results of observations through a light microscope with a magnification of 400X can be seen in Fig. 4A . An image of myofibroblasts expressing COL-1 that yielded a brown positive reaction in the cytoplasm indicates a reaction of COL-1 antigen with monoclonal antibodies (antiCOL-1, ab88147, abcam, USA). Fig. 4B shows the fibroblasts that reacted positively to the most antiCOL-1.

The average description results showed that the group with the highest COL-1 expression was the group receiving treatment with *Stichopus herrmanni* nanoparticle gel with a concentration of 0.27% (SH2), while the lowest was in the control group (K) ( Fig. 4B ).

The results of the Tukey HSD multiple comparisons in Table 1 reveal that the expression of COL-1 on day 7 has a significant difference between the control group and the SH2–3 group. No significant difference occurred between the control group and the HA group and SH1, and the HA group and the SH1–2-3 group.

## Discussion

The use of nanoparticles for biological applications is intended because the nanometer dimensions and large surface area will provide opportunities to bind to more receptors. The development of nanoparticles with specific receptors that are more easily recognized, explicitly recognized, and signaled by macrophages is a critical factor for targeting these specific receptors to become active. 15

The manufacture of nanoparticles from *Stichopus herrmanni* powder was performed using a stainless ball with a high energy milling tool. In the results obtained, it turned out that nanoparticle size has not been obtained in a shorter milling time, even though the time has been added. However, drying using the freeze-drying method facilitated the milling process, so there was a significant difference in the milling process with the addition of time. This result was due to the physical properties of high-molecular *Stichopus herrmanni* powder that has a high modulus. The milling process is quite long, but small diameter ball milling can facilitate the process of changing particles. The diameter of the ball milling was small because the surface area in contact with the test material was greater, so the particle obtained was smaller than before. 16

The nanosize of solid particles ranges from 10 to less than 1,000 nm. However, the preferential size for nanomedical applications is less than 200 nm. 17 In a review conducted by Goyal, 18 the manufacture of nanoparticles on HA materials that have been developed ranges from 486 to 534 nm. In this study, the expected size could be achieved of 153.5 nm using active time, rest time, and stirring for 3 × 30 minutes with stainless ball size of 2 mm.

HA is a naturally occurring substance found in the body. HA plays an essential role in processes such as cell differentiation and has been widely used clinically as an ingredient in regenerative medicine. HA plays a role in the early stages of wound healing as a unique temporary structure to facilitate the diffusion of nutrients at the injury site. 19 HA has a specific receptor (CD44) located on the surface of cell membranes responsible for the migration of fibroblasts to the wound area from the surrounding tissue. CD44 then triggers a signaling cascade that aids in the process of cell growth and cell motility. 20 Hyaluronan receptors, known as receptors for hyaluronan-mediated motility (RHAMM), are distributed on the cell surface or intracellularly in the cytoplasm or nucleus. RHAMM is also highly expressed on the surface of fibroblasts and has been shown to stimulate fibroblast proliferation *in vivo* directly. 21 This was evidenced by the presence of fibroblasts on day 4 after injury in the control and HA groups. It can be maximized, although there is a relatively high increase in the number of fibroblasts.

This nanosize is made to be able to penetrate challenging-to-reach tissues. 22 The nano-size penetration facilitates the entry of binding to the specific receptor for HA, the CD44. It was proven in this study that the group treated with *Stichopus herrmanni* nanoparticle gel showed significant results in increasing the number of fibroblasts. This increase was optimal at a concentration of 0.27% (SH2), equivalent to a pure 0.2% HA content. The increase in concentration was not proportional to the increase in fibroblasts in the SH3 group. In Sari's research, 11 it was found that the presence of chondroitin sulfate and keratin sulfate contained in *Stichopus herrmanni* could decrease the ability of CD44 to bind HA, so that the ligands on cell membranes were reduced, which had an impact on stimulation of cell signaling.

As healing time increases, fibroblasts in the wound bed will become more active by becoming myofibroblasts contributing to collagen synthesis and contraction at the end of the stage. 23 This differentiation process is seen in the results where when the number of fibroblasts increases in the control group, the wound contraction process starts to occur in the treatment group ( Fig. 2B ). The differentiation of fibroblasts to myofibroblasts causes the results of observations of fibroblasts in all groups to show no significant differences ( Table 1 ).

Comparison of statistical results obtained from observations on day 4 and day 7 revealed no significant difference in the control group, HA and SH1, while the SH2 and SH3 groups showed a significant difference with a decrease in the number of fibroblasts due to the transition to myofibroblast form.

Induction of HA at CD44 and RHAMM receptors stimulates HABP1 expression and catenin internalization in endothelial cells. Induction of HA increases the initial formation of blood vessels, actin cytoskeleton rearrangement, cell migration, and adhesion, along with overexpression of the angiogenic factors Vascular Endothelial Growth Factor A ((VEGFA)) and Vascular Endothelial Growth Factor Receptor 1 (VEGFR1) in endothelial cells. 24 This was demonstrated in this study by the significant difference between the control and treatment groups, where there was no significant difference between the treatment groups. The same results were found in the observation of the blood vessels.

During the proliferative phase, fibroblasts secrete extracellular matrix in three stages. In the first stage, fibroblasts will secrete type 3 collagen in the wound bed and are assisted by blood vessels to supply oxygen. Furthermore, wound contraction occurs in the presence of myofibroblasts. The final stage is wound closure with rapid migration of epithelial cells to re-epithelialize. 19

The final stage of the wound-healing process is the maturation phase. The type III collagen formed in this phase is replaced by type I. This change process continues until the healing ends and reaches 80% of the entire tissue, which then becomes mature and has good tissue tensile strength. 25 26 Along with the results of observations of fibroblasts and blood vessels, the expression of type I collagen in this study was sufficient to prove that the group treated with *Stichopus herrmanni* nanoparticle gel showed a relatively high expression of COL-1 where statistically, there was a significant difference between the control group and SH2–3 ( Table 1 ). This result was due to the role of HA with nanoparticle size in accelerating the healing process until it reached the remodeling stage by observing COL-1 expression.

## Conclusion

This study concludes that the administration of *Stichopus herrmanni* nanoparticle gel can accelerate the ulcer healing process. The observation of fibroblasts and blood vessels on day 4 and the observation of blood vessels and COL-1 expression showed a significant increase compared with the control group and HA. The statistical analysis results concluded that *Stichopus herrmanni* nanoparticle gel with a concentration of 0.27% was the most effective in accelerating ulcer healing.
